# Experimental Insights into Free Orthogonal Cutting of Stellite

**DOI:** 10.3390/ma18050921

**Published:** 2025-02-20

**Authors:** Miroslav Gombár, Marta Harničárová, Jan Valíček, Milena Kušnerová, Hakan Tozan, Rastislav Mikuš

**Affiliations:** 1 Department of Machining Technology, Faculty of Mechanical Engineering, University of West Bohemia, 301 00 Pilsen, Czech Republic; gombar@fst.zcu.cz; 2 Institute of Electrical Engineering, Automation, Informatics and Physics, Faculty of Engineering, Slovak University of Agriculture in Nitra, 949 76 Nitra, Slovakia; jan.valicek@uniag.sk; 3 Department of Mechanical Engineering, Faculty of Technology, Institute of Technology and Business in České Budějovice, 370 01 České Budějovice, Czech Republic; kusnerova.milena@mail.vstecb.cz; 4 College of Engineering and Technology, American University of the Middle East, Egaila 54200, Kuwait; hakan.tozan@aum.edu.kw; 5 Institute of Design and Engineering Technologies, Faculty of Engineering, Slovak University of Agriculture in Nitra, 949 76 Nitra, Slovakia; rastislav.mikus@uniag.sk

**Keywords:** orthogonal cutting, Stellite 6, wear, surface roughness, HP/HVOF

## Abstract

The effectiveness of a machining process can be determined by analysing the quality of the generated surface and the rate of tool wear. Stellite is highly challenging to machine, which is why it is primarily processed through grinding methods. This study concentrates on the impact of cutting parameters and tool wear (*VB_b_*, *KB_b_*) on the created surface roughness surface (*Rt*, *Ra*, *Rz*) during the orthogonal cutting of Stellite 6, which is overlaid on a steel surface, C45, prepared by means of HP/HVOF (JP-5000). The results indicate that the dominant influence on the change in the total roughness profile height value (*Rt*) is the mutual interaction of cutting speed and depth of cut at 16% (*p* < 0.000). The greatest impact on the change in the mean arithmetic deviation of the roughness profile (*Ra*) value is the interaction of cutting speed, tool front angle, and depth of cut with a 15% share (*p* < 0.000), as well as on the change in the *Rz* value (15%) and tool wear *VB_b_* (25%). This investigation lays the groundwork for potentially substituting the processing of flat surfaces with hardened layers created by thermal spraying (such as Stellite 6) with grinding or methods that offer greater efficiency from both economic and technological perspectives.

## 1. Introduction

The increasing use of Stellite alloys in various applications has led to a high demand from the materials industry for diverse testing data to acquire further insights into the feasibility of machining these materials. Nonetheless, the research literature indicates that there has been limited exploration into the machinability characteristics of Stellite alloys, with even the database maintained by Kennametal Stellite Inc. highlighting a scarcity of these data [1,2,3]. Cobalt-based alloys are the most difficult to machine. They are rated as having poor machinability. Whenever possible, superalloys should be machined with positive and sharp cutting edges [4]. Geometric shapes of cutting tools that are appropriate for austenitic stainless steel can also be effective on HRSA (heat-resistant superalloys). Nevertheless, reputable producers will feature additional shapes tailored specifically for these demanding materials in their product range [4,5]. Stellite is a type of cobalt alloy commonly utilised for hardfacing [6]. It consists of carbides within a cobalt–chromium matrix, providing it with high levels of hardness, strength, corrosion resistance, and wear resistance [7]. These properties remain intact even at elevated temperatures. Additionally, molybdenum and tungsten are key elements in the alloy, further enhancing its hardness and strength [8,9,10]. Based on cobalt and chromium, these materials possess a high melting point and find application in steam turbines for coatings and hardfacing. The incorporation of elements like nickel enhances the stability of the FCC matrix lattice, while the addition of boron reduces the alloy’s melting point. Stellite alloys are non-magnetic and typically associated with high corrosion resistance and, as with many alloys, they are adaptable and can be refined for a range of specific applications. Non-magnetic materials enhance corrosion resistance through interconnected mechanisms. A uniform microstructure is essential for protective layers that safeguard substrates. Minimising stress concentrations enhances durability [11,12]. Understanding these aspects is crucial for optimising material performance and ensuring reliability across applications. Various production methods are employed to create Stellites, including casting and powder formation. Stellite alloys are mostly produced using the sintering technique. Sintered materials are commonly utilised in various deposition methods for coating a substrate. The forming or processing of these materials typically involves non-traditional machining methods or grinding processes because of their challenging machinability and distinct characteristics [13,14,15]. Machining hard materials presents challenges that always require the search for new methods and tools. Stellite, especially Stellite 6, is one such material that has received a lot of attention because of its high hardness and good resistance to wear and rust. Even though these properties are beneficial, they make standard machining methods more difficult, highlighting the need to improve cutting parameters and methods [16].

Numerous studies have shown that conventional machining techniques often result in accelerated tool wear and unsatisfactory surface finishes, as demonstrated in [17,18].

Stellites come in numerous alloy variations, with different element ratios tailored to specific applications. Stellite 6 and Stellite 21 (SAE AMS 5894D-2016) [19] are commonly used for hardfacing purposes. Within the category of cobalt-based alloys, Stellite alloys stand out as they are specifically engineered to provide high resistance to abrasion. This is attributed to their increased carbon content compared to other cobalt alloys. As a result of a significant volume percentage of Cr_3_C or Mo_2_C carbides, the machinability of Stellite alloys is notably challenging. These alloys are better suited for grinding processes utilising diamond or silicon carbide abrasives. In certain scenarios, machining Stellite using tools equipped with cubic boron nitride insert cutters with tailored coatings may be feasible. Stellite is frequently used as a coating that is deposited by the application of high-velocity combustion or vacuum plasma spraying [20,21,22]. There is still a lack of established machining techniques and processes for Stellite alloys. As Stellites are highly valuable and useful, it is essential to establish appropriate technological procedures for the machining of Stellites. It is hypothesised that the inadequate machined surface of Stellite hard-faced products facilitates corrosion propagation through surface roughness valleys, which ultimately leads to premature failure. Optimal machining of hard-to-machine materials requires a finely tuned system: tool–machine–workpiece. Currently, there are few published publications on the machining of Stellite. The study of free orthogonal cutting of Stellite alloys has changed a lot, mostly because of the material’s tough features and its various uses in industry. In earlier studies, the aim was to learn about basic machining factors like cutting speed, feed rate, and tool wear. Researchers created initial models that used Taguchi methods to improve these factors, showing better surface finish and tool lifespan when working with Stellite alloys [23]. Once these basics were set, further studies in the late 1990s and early 2000s aimed to improve prediction models for wear mechanisms specific to Stellite 6.

Researchers have used finite element methods (FEM) to model heat generation and stress distribution during cutting, clarifying the thermal dynamics involved in machining [24,25]. These simulations not only support experimental results but also enable predictions about tool life and wear patterns, which are crucial for effective machining strategies. Moreover, the use of soft computing techniques like neural networks is becoming more popular, allowing for better predictions based on large datasets from empirical studies [26]. There are a number of metalworking processes like turning, drilling, milling, boring, reaming, and various others providing significant added value. Advancements in the metalworking sector have focused on enhancing production efficiency and performance through a deeper comprehension of methods to enhance quality, decrease production time, and lower operational expenses. The intricate nature of metalworking procedures is evident. Despite extensive research conducted over the past century, there remain certain knowledge gaps that hinder a complete comprehension of the interplay between the cutting tool and the material being worked on, along with other process variables [27].

The elements, together with carbon, form very hard carbides in the structure of the coating and cause intense dulling of the cutting edge in the machining process. It is for these reasons that grinding technology [16,28] is nowadays mainly used to reduce the surface roughness of tooling. Grinding of thermally applied coatings is a relatively time-consuming technological operation due to the small depths of cut. There are a number of complications associated with the process of grinding thermally applied coatings, which must be taken into account. Problems of a technological nature can include an incorrectly ground grinding wheel and the consequent failure to achieve the required roundness of the workpiece. If the filter is not functioning properly, small particles of the material being removed can be re-entrained into the cutting process by the cutting fluid. Possible problems can also arise from improper grinding wheel selection, where the grinding grain size, choice of abrasive material, and bond must be taken into account. Due to the high speed of the grinding wheel, possible vibration generation also needs to be addressed [28].

Grinding is the primary method for effectively machining Stellite, mainly due to its significant hardness [29]. Recent advancements in cutting tool materials and designs have facilitated more efficient orthogonal cutting approaches, particularly for hard materials, as emphasised in [29]. The intricate correlation between cutting parameters and surface roughness has been extensively explored in various materials, with key factors such as cutting speed and depth of cut emerging as focal points, as discussed in the research of Zhang et al. in [30]. However, there is a noticeable lack of research specifically targeting Stellite 6, highlighting a significant gap that this study aims to meticulously investigate and address.

When grinding thermally applied coating, it is necessary to remember that the thermally applied coating is not a volumetrically homogeneous material but that the structure of the coating is made up of individual platelets of fused particles which form a lamellar structure. Another problem that can arise when the cutting conditions are not properly adjusted is the generation of high temperatures at the cutting point. The high temperatures cause structural and phase changes and, thus, tensile residual stresses can occur as the workpiece cools [31,32,33]. These facts then contribute to the formation of cracks and, hence, a reduction in the service life of machine components [34]. Due to the above-mentioned problems that arise in the grinding of thermally applied coatings, and, at the same time, desire to improve the economics of surface treatment of thermally applied coatings, there has been an effort to replace this process with other chip machining processes.

Additive manufacturing utilising WAAM (Wire Arc Additive Manufacturing) and laser cladding is an effective method for fabricating components and coatings from Stellite 6 with outstanding mechanical properties. The selection of suitable machining and coating parameters is key to achieving the desired surface roughness and wear resistance properties [35,36,37].

In the presented work, the machinability of Stellite 6 (SAE AMS 5894D-2016) material was analysed using free orthogonal cutting of Stellite 6. The objective of this study is to examine the detailed machining processes of Stellite 6 in order to determine the optimum cutting parameters based on the best value of surface roughness. Additionally, comprehensive statistical analyses are often lacking, which are vital for identifying key operational limits. To summarise, while notable progress has been made in understanding the complexities of free orthogonal cutting of Stellite, the journey ahead requires continued exploration and refinement in methods. Addressing the identified limitations and broadening the focus of future research can help establish best practices that not only enhance performance but also push the boundaries of achievable results in machining hard materials. Ultimately, the insights reviewed here provide a solid foundation for further academic inquiry and industrial application, ensuring that the challenges of machining Stellite are met with innovative and effective solutions.

## 2. Materials and Methods

### 2.1. Material

Stellite 6 can be in bulk form as a compact material [38,39], or in the form of a spray applied by TIG welding [40], PTA deposition [41], laser deposition [42,43] or HVOF deposition [44]. The microstructure of Stellite 6 formed by HVOF spraying method consists of Co-rich dendrites with a surface-centred cubic (FCC) crystal structure surrounded by a lamellar Co alloy and a carbide phase formed during its solidification process.

In Stellite 6 alloys, Cr provides high resistance to oxidation and corrosion; at the same time, it contributes to the high wear resistance to carbide formation, M_7_C_3_ and M_23_C_6_. Refractory metals, such as Mo and W, represent elements that harden solid solution, thus contributing to strength enhancement by precipitation hardening of MC and M_6_C carbides and intermetallic phases such as Co_3_(Mo, W). In addition, alloying with the addition of Ni, C, and Fe promotes the stability of the Co-rich FCC structure, which is then stabilised at high temperatures up to the melting point (1495 °C), whereas Co, Mo, and W tend to stabilise at low temperatures into a HCP crystal structure that is stable up to 417 °C. Samples of steel (C45) (EN 10083-2:2006) [45] in normalised condition with Stellite 6 coating, moulded by applying the HP/HVOF JP 500 method, were used in the experimental validation. The spray material (a Co-based gas atomised powder (similar to Stellite 6)), with nominal particle size distribution −53 + 20 μm and nominal chemical composition (in mass %) 28.5Cr, 4.4 W, 1.5Fe, 1.5Ni, 1.1C, 1Si and balance Co, was chosen for this study (Co-based 2637–02, Höganäs, Bruksgatan, Sweden) is typically delivered in the form of small particles to a spraying device, where it is heated and accelerated towards the substrate. The molten particles gradually deform into scales and lamellae upon contact with the substrate surface and solidify rapidly in the form of a disk. The repeated impact of the particles then produces a coating with a lamellar microstructure with specifically anisotropic properties. In addition to the deformed particles, the microstructure of the coating also contains numerous defects, such as oxides, pores, and impurities. The presence of these defects reduces the mechanical properties of the coating, and, at the same time, the nature of the defects defines the quality of the coating itself. The basic parameters of the coating applied to the test specimens (26 pc) are given in Table 1. The conditions before spraying were as follows: grain size 0.8–1 mm (F22); substrate surface roughness *Ra* = 8 μm; FST 484.074 Stellite 6 component based on a powder blend with a nominal composition of 28% Cr, 5% W, 1.2% C, 1% Si.

**Figure 1:**
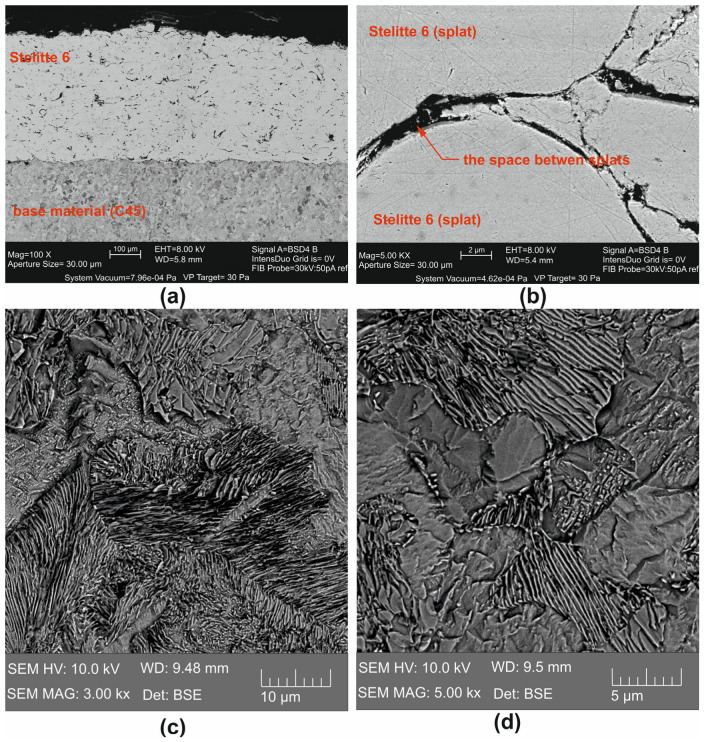
Microstructure of the Stellite 6 machined material: (**a**) interface between the base material and the coating; (**b**) gap between the coating deposits; (**c**,**d**) ferritic and pearlitic microstructures of C45.

From the microstructure of Stellite 6 spray (Figure 1) obtained by scanning electron microscopy Zeiss Auriga Cross Beam SEM-FIB (Carl Zeiss AG, Oberkochen, Germany) it is possible to observe a relatively low degree of porosity (Figure 1a) and gaps between the individual platelets of the coating (Figure 1b). The microstructure of Stellite 6 was revealed by etching in 3% Nital. The ferritic and pearlitic microstructures of C45 are clearly defined in Figure 1c,d.

**Figure 2:**
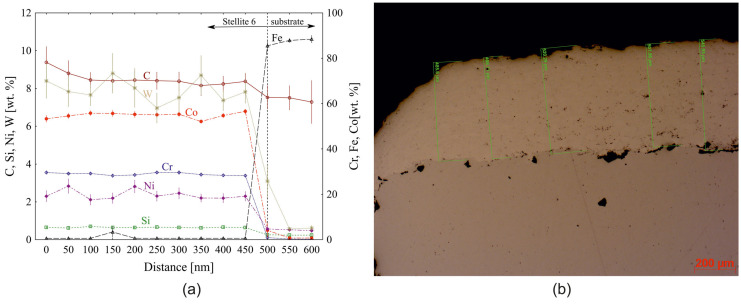
Chemical composition of the (**a**) coating; (**b**) the thickness of Stellite 6 coating.

In Figure 2a, we present the results of Energy Dispersive Spectrometry (Zeiss Auriga Cross Beam SEM-FIB) analysis for the determination of the chemical composition of the machined Stellite 6 material and substrate as a function of distance from the coating surface, and, in Figure 2b, the measurement of the coating thickness itself. The profile of the coating, as can be seen from Figure 2b, is convex in nature, with an average thickness of 502 ± 18 µm.

**Figure 3:**
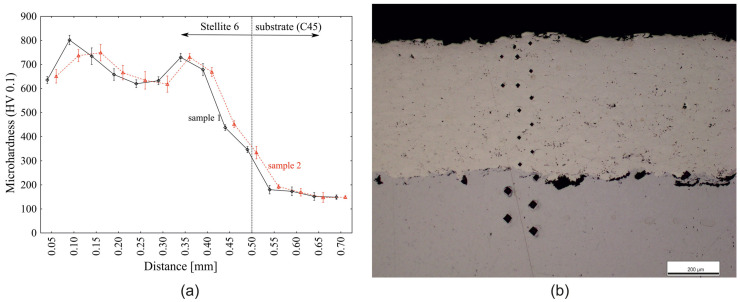
Microhardness values (HV 0.1) on Stellite 6: (**a**) variation in HV 0.1 as a function of distance from the surface of the coating; (**b**) microhardness measurement record.

In order to ensure the machining constancy in terms of the actual tooling on the individual specimens, 2 specimens (specimen 1, specimen 2) were randomly selected from the set of 26 specimens on which the microhardness HV0.1 (0.9807 N, 10 s) (DuraScan 50, Zwick Roell) (Zwick Roell, Ulm, Germany) was evaluated (Figure 3). The average microhardness value of sample 1 was 659 ± 22, with the variation in distance from the coating surface varying from a value of 636 ± 6 at 0.05 mm to 438 ± 5 at a distance of 0.45 mm from the coating surface to a maximum value of 802 ± 8 at a distance of 0.10 mm from the coating surface. Sample 2 achieves an average microhardness value of 656 ± 19. Thus, the difference between the mean microhardness value of sample 1 and sample 2 is 2.350 ± 3.850 and is not significant at the α = 5% significance level in terms of Tukey’s test (*p* = 0.229). On this basis, it can be said that the randomly selected samples are homogeneous in terms of microhardness, and it is therefore assumed that the other samples intended for the implementation of the experimental validation will also statistically show the same microhardness values HV 0.1.

Porosity measurements of Stellite 6 (measured using an Olympus Lext OLS 3100 (Olympus Corporation, Tokyo, Japan) confocal microscope by phase analysis) were carried out on two selected samples (sample 1, sample 2). Sample 1 achieves an average porosity value of 0.557 ± 0.032% and sample 2 achieves an average porosity value of 0.563 ± 0.038%. The difference between the mean porosity values of sample 1 and sample 2 is −0.005 ± 0.041% and is not significant (*p* = 0.827) in terms of Tukey’s test at the selected significance level *α* = 0.05. From the point of view of microhardness and porosity, both samples (sample 1, sample 2) can be considered identical and, on the basis of the analysis carried out, it is also possible to accept the assumption that the other samples, the properties of the Stellite 6 thermally applied coating, will not differ from each other in a statistical sense.

### 2.2. Machine, Tool, Workpiece

**Figure 4:**
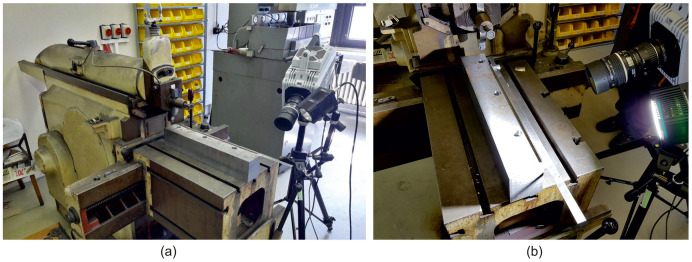
Implementation of experimental verification: (**a**) a horizontal trimming machine, (**b**) a more detailed view of the machine’s workspace.

In the experimental verification, a horizontal trimming machine (Figure 4) with the designation Strigon GH560/U (Strigon, Svitavy, Czech Republic) was used, whose basic technical specifications are given in Table 2.

**Figure 5:**
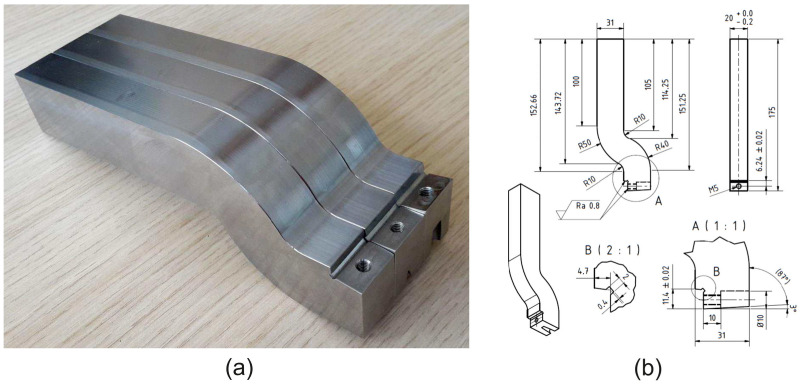
Machining tool: (**a**) tools for different variations of face angles, (**b**) technical drawing of the tool for *γ* = 0°.

For the implementation of the experiment, shaping blades (Figure 5a) were produced in three basic variations of the tool face angle values used (Table 3). The shaping tool is similar in shape to the turning tool, the tangential turning tool. During shaping, the tool is subjected to a relatively large impact at each stroke on the first contact with the workpiece. These impacts, together with the typically higher shaping tool lining, can cause intense vibration and damping of the cutting tool. These two phenomena have a major impact on the stability of the cutting process, which can ultimately affect the quality and accuracy of the machined surface. The chosen strategy to eliminate these undesirable phenomena was to design the tool in a bent state (Figure 5a,b). Bending of the tool reduces the tool’s degree of damping and the change in tool trajectory during damping. In this design, the shaping blade is made so that the tool’s tip aligns with the base of the clamping section. The shaping tools were made of 42CrMo4 steel with the following properties: *R_e_* = 750 MPa, *R_m_* = 1000–1200 MPa. These mechanical properties are typical for this steel in the quenched and tempered condition. This material is used to manufacture turning chucks. Due to the experimental conditions (Table 3), the shaping tool was chosen as a tool with a replaceable VBD. For the experiment, a VBD with the designation SCMW 120408 (Iscar ČR, s.r.o, Prague, Czech republic) made of sintered carbide IC20 (Iscar ČR, s.r.o.) was chosen. The dimensions of the VBD were *l* = 12.7 mm, *S * = 4.76 mm, *r * = 0.8 mm. In order to eliminate the effect of VBD wear on the change in the evaluated surface quality parameters, a new VBD was used for each experiment.

### 2.3. Technological Conditions of the Experiment

In order to analyse the machining process of the Stellite 6 thermally applied coating, the basic parameters of the machined surface roughness (*Rt*, *Ra*, *Rz*) and the wear characteristics of the face and back of the cutting tool were evaluated. The cutting speed, the depth of cut, and the tool face angle were used as input variables (Table 3).

At the same time, the following factors were held constant throughout the experiment:

- tool lining: 90 mm
- cutting insert (VBD): SCMW 120408 I20
- material to be machined: Stellite 6
- tool back angle: *β* = 90°
- cooling: no

## 3. Results and Discussion

### 3.1. Analysis of Roughness Parameters of the Machined Surface

The roughness of the unmachined thermally applied coating is influenced by the structure of the coating itself, which consists of fused or partially fused deformed particles (splats). It also contains a large number of inclusions, impurities, pores, and oxides. The actual surface roughness of the thermally applied coating is also influenced by the deposition method and the type of additive. In the case of powder material, the roughness depends on the chemical composition and the particle size. As part of the analysis, the profile roughness parameters were measured on a Hommel Etamic T8000 (HOMMEL CS, Teplice, Czech Republic), which measures surface roughness by touching the measuring tip. In accordance with DIN EN ISO 4288:1998 [46], a tip size of 2 µm was chosen. The measurement was carried out on seven sample locations with a constant measured length of 4.8 mm. The measurement feed rate was set to *v_t_* = 0.50 mm·s^−1^.

**Figure 6:**
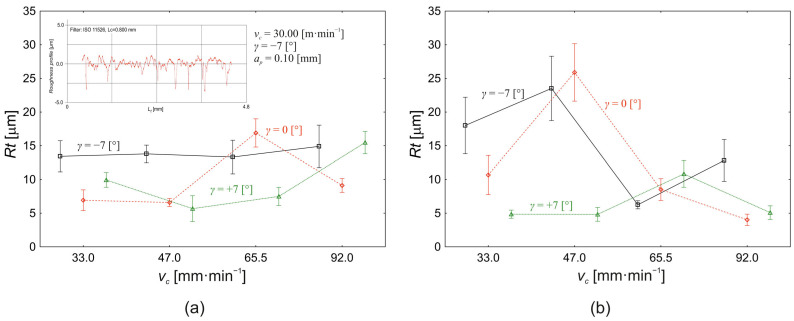
Effect of the cutting speed (*v_c_*) and the tool face angle (*γ*) on the change in the value of the total roughness profile height *Rt*: (**a**) *a_p_* = 0.10 mm; (**b**) *a_p_*= 0.30 mm.

The variation in the total roughness profile height (*Rt*) as a function of the cutting speed value (*v_c_*) for each tool face angle value (*γ*) used in the experiment is shown in Figure 6a (*a_p_* = 0.1 mm) and in Figure 6b (*a_p_* = 0.30 mm). For the depth of cut *a_p_* = 0.10 mm (Figure 6a), based on the analysis carried out, it can be said that the effect of the cutting speed on the change in the value of the total roughness profile height (*Rt*) was 11%. Since technological machining processes are classified as stochastic processes with a large number of acting factors and, at the same time, in accordance with the measurement theory, it is necessary to consider the variability of the measured values due to the influence of the lack of measurement, the use of statistical procedures seems to be the correct approach. The effect of the cutting speed on the change in the value of the investigated response (*Rt*) is significant (*p* = 0.013) at the chosen significance level *α * = 0.05 in terms of Fisher’s analysis of variance (ANOVA). At the same time, there was a statistically significant effect (*p* = 0.003) of the tool face angle (*γ*) at the 11% level, as well as the interaction between the cutting speed and the face angle at the 24% level (*p* = 0.003) on the change in *Rt* value. At the same time, it should be said that other factors not considered in the experiment also influenced the change in *Rt* value, and their share is 54%.

Figure 6a shows that, when the tool face angle is used at *γ* = −7°, the effect of the cutting speed on the change in the value of the total profile roughness (*Rt*) is negligible. The values of *Rt* vary at *γ* = −7° from 13 ± 6 µm at a cutting speed of 33.0 m·min^−1^ to a value of 15 ± 9 µm at a cutting speed of 92.0 m·min^−1^. Thus, the total change in *Rt* value between the minimum and maximum value of the cutting speed used is 11%. At the tool face angle *γ* = 0°, the value of the variable under study (*Rt*) varies minimally in the interval of cutting speeds 33.0 m·min^−1^ (7 ± 4 µm) to 47.0 m·min^−1^ (7 ± 2 µm). A significant increase in the studies response (*Rt*) is observed at a cutting speed of 65.5 m·min^−1^ (17 ± 5 µm) followed by a sharp decrease to *Rt * = 9 ± 3 µm for a cutting speed of 92.0 m·min^−1^. The change in *Rt* value at the tool face angle *γ* = +7° is quite different in nature. Here, we observe a decrease in the observed response (*Rt*) from a value of 9 ± 3 µm at *v_c_* = 33.0 m·min^−1^ to a value of 5.7 ± 4.9 µm at *v_c_* = 47.0 m·min^−1^. By further increasing the cutting speed, the *Rt* value increases to a level of 8 ± 3 µm at *v_c_* = 65.5 m·min^−1^ to its maximum value at 16 ± 4 µm at *v_c_* = 92.0 m·min^−1^. At the same time, however, according to Scheffe’s test [47] of mutual differences of average values, it is possible to conclude that, for depth of cut *a_p_* = 0.10 mm, there are statistically significant differences in the mean values of the total profile roughness (*Rt*) between tool face angles *γ* = −7° and *γ* = 0° (4 ± 1 µm, *p* = 0.021) and between tool face angles *γ* = −7° and *γ* = +7° (4 ± 1 µm, *p* = 0.009). A significant difference in mean values of the investigated variable *Rt* was not confirmed (*p* = 0.985) when instruments with the face angle *γ* = 0° and *γ* = +7° were used.

Using the depth of cut *a_p_* = 0.30 mm (Figure 6b), it is possible to observe a different pattern of change in the total roughness profile height (*Rt*) as a function of the change in cutting speed (*v_c_*) at three different tool face angles (*γ*). The effect of the cutting speed on the change in the response value (*Rt*) is 21% (*p* < 0.000), the effect of the tool face angle is 15% (*p* < 0.000), and the effect of the interaction between the cutting speed and the tool face angle is 29% (*p* < 0.000). Logically, by increasing the influence of the controlled factors used on the change in *Rt* value, the influence of the other variables not considered in the experiment drops to 35%, compared to the proportion of the effects at 0.10 mm depth of cut. At a tool face angle of *γ* = −7°, increasing the cutting speed from a value of *v_c_* = 33.0 m·min^−1^ to a value of *v_c_* = 47.0 m·min^−1^ increases the average value of the total roughness profile height from a value of 18 ±1 3 µm to a value of 24 ± 13 µm. A similar trend in the change in the value of the variable *Rt* within the cutting speed interval of 33.0 m·min^−1^ to 47.0 m·min^−1^ is also evident for the tool face angle *γ* = −7°. In this case, the conditional value of the total profile roughness height increases from 11 ± 8 µm to a value of 26 ± 12 µm. For both reported face angle settings, when the cutting speed is increased to a value of *v_c_* = 65.5 m·min^−1^, the average *Rt* response value decreases to a value of 6.3 ± 1.6 µm (*γ* = −7°) and 9 ± 4 µm (*γ* = 0°), respectively. The difference in the *Rt* response value occurs at cutting speeds *of_c_* = 92.0 m·min^−1^, and this is the increase in the conditional *Rt* value to 13 ± 9 µm and a further decrease in the *Rt* value to 4.017 ± 2.169 µm at the tool face angle *γ* = −7° and *γ* = 0°, respectively. At tool face angle *γ* = −7°, the value of the observed *Rt* response changes when the cutting speed value increases from *v_c_* = 33.0 m·min^−1^ (5 ± 2 µm) to *v_c_* = 47.0 m·min^−1^ (5 ± 3 µm). A further increase in cutting speed to a value *v_c_* = 65.5 m·min^−1^ also increases the *Rt* value to a level of 11 ± 5 µm with a subsequent decrease in the observed *Rt* response to a value of 5 ± 3 µm for a cutting speed *v_c_* = 92.0 m·min^−1^.

If we also consider the effect of the depth of cut (*a_p_*) on the change in the value of the total roughness profile roughness (*Rt*) in terms of the analysis performed (Figure 6), we conclude that the effect of the other controlled factors (*v_c_, γ*) changes significantly compared to the partial conclusions. The effect of cutting speed on the change in the observed response value *Rt* is 2% (*p* = 0.046), the effect of tool face angle is 11% (*p* < 0.000), and the effect of depth of cut is 0.1% (*p* = 0.871). Thus, depth of cut as a main effect is not a significant factor that affects the change in the value of the total profile roughness. However, it is possible to define significant interactions that have a significant effect on the change in the value of *Rt*. The interaction of cutting speed and tool face angle has an overall contribution to the change in the response value (*Rt*) of 14% (*p* < 0.000), the interaction of the cutting speed and the depth of cut has an overall contribution of 16% (*p* < 0.000), the interaction of the tool face angle and the depth of cut 2% (*p* = 0.001), and the interaction of the cutting speed, tool face angle, and depth of cut 13% (*p* < 0.000). A very important conclusion can be drawn from the above results. Technological machining processes must be understood and analysed not only in terms of the influences acting as main, separate effects, but also in terms of the interactions between factors that may have a significantly greater influence on the observed response.

**Figure 7:**
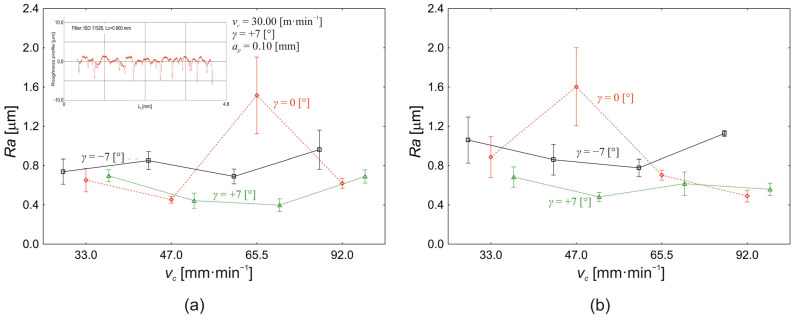
Effect of the cutting speed (*v_c_*) and the tool face angle (*γ*) on the change in the value of the mean arithmetic deviation of the roughness profile *Ra*: (**a**) *a_p_* = 0.10 mm; (**b**) *a_p_* = 0.30 mm.

A graphical representation of the variation in the mean arithmetic deviation in the roughness profile (*Ra*) as a function of the change in the cutting speed (*v_c_*) and the tool face angle (*γ*) is shown in Figure 7a for *a_p_* = 0.10 mm depth of cut and in Figure 7b for *a_p_* = 0.30 mm depth of cut. For the depth of cut at *a_p_* = 0.10 mm (Figure 7a), there was no significant effect of the cutting speed as a main, independent effect (5%, *p* = 0.094) on the change in the value of the investigated response *Ra*. The effect of the tool face angle on the change in *Ra* value is 8% (*p* = 0.013), and, at the same time, the effect of the interaction between the cutting speed and the tool face angle was significant (*p* < 0.000) with 29% effect on the change in the value of roughness *Ra*.

Figure 7a shows that the surface roughness *Ra* at the tool face angle *γ* = −7° varies from a value of 0.738 ± 0.328 µm at the cutting speed *v_c_* = 33.0 m·min^−1^ to a value of 0.961 ± 0.487 µm at the cutting speed *v_c_* = 33.0 m·min^−1^. Thus, the total change in the roughness value *Ra* between the minimum and maximum value of the cutting speed used is 30%. After changing the tool face angle to *γ* = 0°, the surface roughness value *Ra* changes from a value of 0.653 ± 0.293 µm at a cutting speed *v_c_* = 33.0 m·min^−1^ to a value of 0.453 ± 0.087 µm at a cutting speed *v_c_* = 47.0 m·min^−1^. Thus, increasing the cutting speed within the above interval results in a decrease in *Ra* value by 31%. However, within Figure 7a, we observe a sharp increase in the surface roughness value *Ra* at cutting speed *v_c_* = 47.0 m·min^−1^, namely, at a level of 1.515 ± 1.006 µm followed by a decrease in the *Ra* value to a level of 0.620 ± 0.125 µm at the cutting speed *v_c_* = 92.0 m·min^−1^. At the third tool face angle setting level, namely *γ* = +7°, the surface roughness *Ra* value changes from a value of 0.697 ± 0.151 µm at a cutting speed *v_c_* = 33.0 m·min^−1^ to a value of 0.690 ± 0.165 µm at a cutting speed *v_c_* = 92.0 m·min^−1^. Figure 7a further shows that the minimum value of surface roughness *Ra* (0.397 ± 0.158 µm) at cutting depth *a_p_* = 0.10 mm was obtained at the tool face angle *γ* = +7° and the cutting speed *v_c_* = 65.5 m·min^−1^. In general, the lowest average surface roughness value *Ra* was obtained in the experiment using a tool face angle of *γ* = +7°, i.e., 0.557 ± 0.158 µm. Changing the tool face angle to *γ* = 0° resulted in an average surface roughness *Ra* value of 0.784 ± 0.241 µm and 0.818 ± 0.139 µm for a tool face angle of *γ* = −7°. However, a significant difference in the mean surface roughness *Ra* values was only demonstrated between the tools with a face angle of *γ* = −7° and the tools with a face angle of *γ* = +7° (*p* = 0.033). For the cutting depth *a_p_* = 0.30 mm (Figure 7b), as in the previous experiment (Figure 7a), there was no significant effect of the cutting speed on the change in the surface roughness value *Ra* as a main effect (*p* = 0.142). The effect of the tool face angle on the change in the surface roughness value Ra is 11% (*p* = 0.004), and the effect of the interaction between the cutting speed and the tool face angle is 21% (*p* = 0.002). Increasing the depth of cut from *a_p_* = 0.10 mm to *a_p_* = 0.30 mm increases the proportion of the effect of the face angle on the roughness value *Ra* by 3% and, at the same time, decreases the proportion of the effect of the interaction of the tool face angle and the cutting speed by 8%. If we also analyse the influence of the depth of the cut itself (*a_p_*) on the change in the surface roughness value *Ra* (Figure 7), the interaction of the cutting speed, the tool face angle, and the depth of cut have the greatest influence on the change in the value of the studied response with a 15% share (*p* < 0.000). The second most significant influence on the change in the roughness value *Ra* is the interaction of the cutting speed and the tool face angle (*p* = 0.002) with 10% contribution, followed by the tool face angle (as the main effect) with 9% contribution to the change in *Ra* value (*p* < 0.000). The interaction between the cutting speed and the depth of cut is also a significant effect, with 5% contribution to the change in *Ra* value (*p* = 0.010). At the same time, it should be said that neither cutting speed nor the depth of cut have a significant effect on the change in *Ra* value as separate main effects.

**Figure 8:**
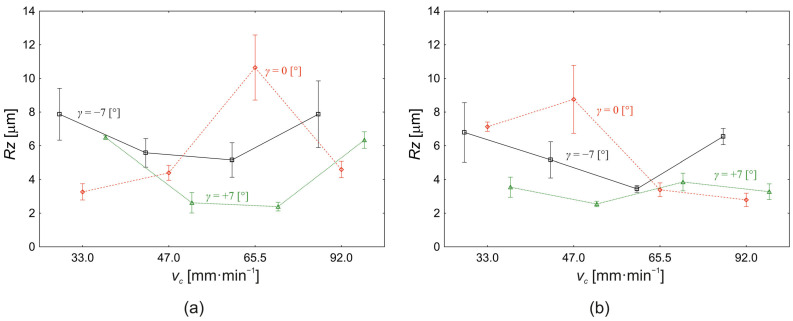
Effect of cutting speed (*v_c_*) and face angle (*γ*) on the change in the value of the largest profile height *Rz:* (**a**) *a_p_* = 0.10 mm; (**b**) *a_p_* = 0.30 mm.

The third surface roughness characteristic evaluated, namely the largest profile roughness height *Rz*, is shown in Figure 8a for the cut depth *a_p_* = 0.10 mm and Figure 8b for the cut depth *a_p_* = 0.30 mm.

**Figure 9:**
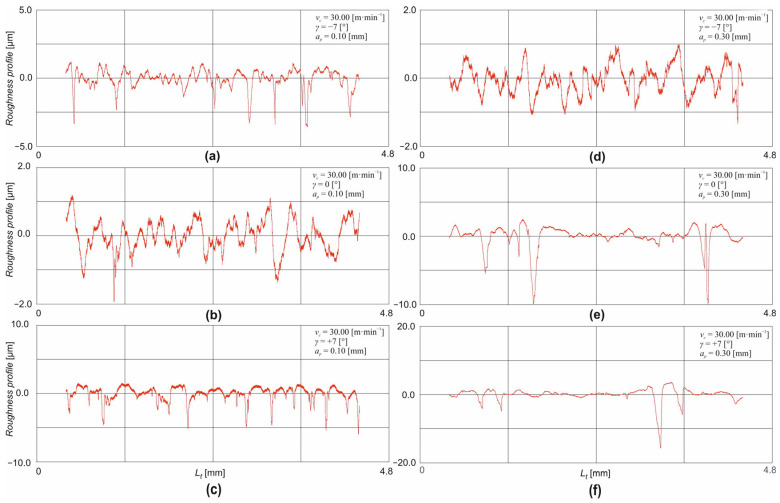
Roughness profile record for the cutting speed *v_c_* = 33.0 m·min^−1^: (**a**) *γ* = −7°, *a_p_* = 0.1 mm; (**b**) *γ* = 0°, *a_p_* = 0.1 mm; (**c**) *γ* = +7°, *a_p_* = 0.1 mm; (**d**) *γ* = −7°, *a_p_* = 0.3 mm; (**e**) *γ* = 0°, *a_p_* = 0.1 mm; (**f**) *γ* = +7°, *a_p_* = 0.1 mm.

**Figure 10:**
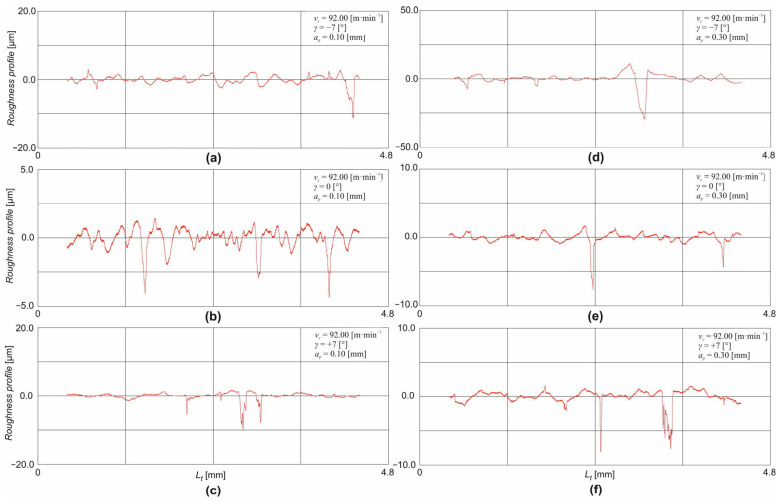
Roughness profile record for the cutting speed *v_c_* = 92.0 m·min^−1^: (**a**) *γ* = −7°, *a_p_* = 0.1 mm; (**b**) *γ* = 0°, *a_p_* = 0.1 mm; (**c**) *γ* = +7°, *a_p_* = 0.1 mm; (**d**) *γ* = −7°, *a_p_* = 0.3 mm; (**e**) *γ* = 0°, *a_p_* = 0.3 mm; (**f**) *γ* = +7°, *a_p_* = 0.3 mm.

From the above figures (Figure 9 and Figure 10), it is clear that the roughness profile of the machined surface is mainly formed by depressions. The machining of thermally applied coatings is accompanied by a change in surface integrity, which includes, for example, the adhesion of the formed coating, the hardness of the coating, and the deformation hardening or residual stresses. Due to the low thicknesses of thermally applied coatings, the main reason being the economic aspect of their formation in the machining process, there is a problem with the size and depth of the affected layer. Due to the aforementioned low thicknesses of the filament coatings, there is a high risk of plastic deformation affecting the base material in the machining process. The induction of plastic deformation to such a depth may result in the cracking or partial tearing of the filament coating. For this reason, small depths of cut are chosen when machining filamentary tooling, but these must be greater than the radius of curvature of the tool tip for the stability of the cutting process. Problems arise in the machining of thermally applied coating primarily for two reasons. The first reason is the adhesion of the wire shot to the base material. The second reason is the high hardness of the wire shot and the high content of difficult-to-machine elements, which impairs machinability and causes intense wear on the cutting tool (Section 3.2). Poor adhesion of the spray to the base material is typical, especially at the edges of the sprayed blank, where the spray loses cohesion with the base material, and, very often, complete delamination of the coating occurs at these locations. Thus, the adhesion of the thermally applied coating to the base material is a very important property of the coating under thermal or mechanical loading. After the actual application of the thermally applied coating, rapid solidification of the additive material occurs, resulting in the formation of tensile residual stresses in the structure. The magnitude of these stresses varies depending on the process parameters during the formation of the coating: the magnitude of the fusion temperature, the velocity and type of carrier gas, the application distance, the type of environment, etc. The overall surface quality and, thus, the adhesion is also largely influenced by the degree of micro-defects formed in the transition region: porosity, impurities, oxides, etc.

The clustering of a large number of these micro-defects, especially in the transition region, leads to the damage of the tooling in the machining process. The specific combination of difficult-to-machine elements creates a heavy load on the cutting tool. Carbides and oxides are present in the structure of the machined tooling and cause severe abrasion of the cutting tool. Regardless of the method of formation of the wire shot, its structure consists of fused particles of a disc shape so that the structure of the wire shot is heterogeneous. In addition to the adhesion of the coating to the base material, the question of the mutual cohesion of the homogeneous particles of the coating, the splats, also arises. It is the combination of the cohesiveness of the splats and the micro-defects (porosity, presence of oxides, and cracks) in the coating that causes the typical shape of the roughness profile, where the surface is formed by the extraction of the splats from the coating, in addition to the cutting process, as can be seen, for example, in Figure 9e,f and Figure 10c–e.

### 3.2. Analysis of Tool Wear Parameters

**Figure 11:**
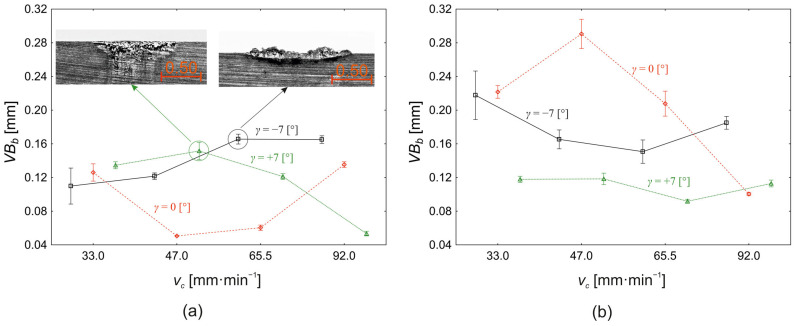
Effect of the cutting speed (*v_c_*) and face angle (*γ*) on the variation in the tool back wear area (*VB_b_*): (**a**) *a_p_* = 0.10 mm; (**b**) *a_p_* = 0.30 mm.

Within the tool back wear analysis, the response parameter observed was *VB_b_*. The variation in parameter *VB_b_* with the cutting speed and the tool face angle is shown in Figure 11a for the depth of cut *a_p_* = 0.10 mm and Figure 11b for the depth of cut *a_p_* = 0.30 mm, respectively. Analysing the tool back face wear for the depth of cut *a_p_* = 0.10 mm (Figure 11a), we conclude that the cutting speed has no significant effect (*p* = 0.143) on the change in *VB_b_* value. The tool face angle contributes 20% (*p* < 0.000) to the change in the tool back wear value, and the interaction between the cutting speed and the tool face angle has a dominant effect on the change in the *VB_b_* value with a contribution of 58% (*p* < 0.000).

For the tool face angle *γ* = −7°, the average tool back wear is 0.141 ± 0.015 mm. However, from Figure 11a, it can be seen that the value of *VB_b_* increases with increasing the value of cutting speed, from 0.110 ± 0.052 mm (*v_c_* = 33.0 m·min^−1^) to 0.166 ± 0.014 mm (*v_c_* = 65.5 m·min^−1^). At the same time, within Figure 11a, it is possible to notice the occurrence of an increase on the tool back surface. By further increasing the cutting speed to the level of *v_c_* = 92.0 m·min^−1^, the value of the tool back wear changes minimally and reaches 0.166 ± 0.011 mm. When the tool face angle is set to the level *γ* = 0°, the nature of the change in the tool back wear value changes. From a value of 0.126 ± 0.025 mm at a cutting speed *v_c_* = 33.0 m·min^−1^, the value of *VB_b_* decreases to a level of 0.051 ± 0.001 mm at a cutting speed *v_c_* =47.0 m·min^−1^. This is followed by a slight increase in the back wear to a level of 0.060 ± 0.008 mm (*v_c_* = 65.5 m·min^−1^), while the difference in this quasi-stationary region is not significant (*p* = 0.999). Increasing the cutting speed to a level of *v_c_* = 92.0 m·min^−1^ again results in a sharp increase in the tool back wear to 0.135 ± 0.008 mm. Changing the tool face angle to a value of *γ* = +7° again yields a different pattern of change in the tool back wear as a function of the change in the cutting speed. From a value *VB_b_* = 0.135 ± 0.010 mm (*v_c_* = 33.0 m·min^−1^), increasing the cutting speed to a level *v_c_* = 47.0 m·min^−1^ increases the wear value slightly to a level of 0.152 ± 0.025 mm. By further increasing the cutting speed, the tool back wear value decreases to 0.121 ± 0.008 mm (*v_c_* = 65.5 m·min^−1^) or to 0.053 ± 0.007 mm at a cutting speed *v_c_* = 92.0 m·min^−1^.

For the depth of cut *a_p_* = 0.30 mm (Figure 11b), the cutting speed (*p* < 0.000) has a significant effect on the change in the back wear (*VB_b_*), with a 14% contribution to the change in *VB_b_* value, in contrast to the conditions shown in Figure 11a. The face angle contributes 37% (*p* < 0.000) to the change in the back wear, and the interaction between the cutting speed and the tool face angle contributes 28% (*p* < 0.000). In addition to the change in the influence of the homogeneous input factors on the back wear value, the depth of cut (*a_p_* = 0.30 mm) also exhibits a different pattern of change in *VB_b_*. For the face angle *γ* = −7°, increasing the cutting speed in the interval from *v_c_* = 33.0 m·min^−1^ to *v_c_* = 65.5 m·min^−1^ results in a gradual decrease in the back wear value from 0.218 ± 0.070 mm to 0.151 ± 0.034 mm. By further increasing the cutting speed to *v_c_* = 92.0 m·min^−1^, the back wear value increases to 0.185 ± 0.019 mm. For a tool face angle of *γ* = 0°, increasing the cutting speed from *v_c_* = 33.0 m·min^−1^ to *v_c_* = 47.0 m·min^−1^ increases the back wear value from 0.222 ± 0.018 mm to its maximum value of 0.291 ± 0.043 mm with a subsequent decrease to a level of 0.100 ± 0.005 mm at a cutting speed of 33.0 m·min^−1^. At a tool face angle of *γ* = +7°, the back wear value ranges from 0.118 ± 0.009 mm to 0.113 ± 0.010 mm with a minimum of 0.092 ± 0.006 mm at a cutting speed *v_c_* = 92.0 m·min^−1^. If we consider the effect of the depth of cut (Figure 11) on the change in tool back wear value, the most significant effect on the change in *VB_b_* is the interaction of the cutting speed, tool face angle, and depth of cut itself with a contribution to the change in the tool back wear at 25%. The second most significant influence on the change in the tool back wear value is the depth of cut (16%), followed by the interaction of the tool face angle and the depth of cut (16%). The tool face angle also has a relatively high influence value on the change in the tool back wear as a main effect (11%). The cutting speed also has the smallest but still significant effect (*p* < 0.000), with 4% effect on the change in *VB_b_* value. Comparing Figure 11a and Figure 11b, we conclude that there are significant differences in the mean values of the tool back wear using cutting depths of *a_p_* = 0.10 mm and *a_p_* = 0.30 mm, respectively. There is a significant difference (*p* < 0.000) in the mean values of tool back wear (−0.062 ± 0.012 mm) between the depth of cut *a_p_* = 0.10 mm (Figure 11a) and *a_p_* = 0.30 mm (Figure 11b) for the cutting speed *v_c_* = 33.0 m·min^−1^. The same statistically significant differences between the used depths of cut are also demonstrated for the cutting speed *v_c_* = 33.0 m-min^−1^ (−0.083 ± 0.014 mm, *p* < 0.000) and for the cutting speed *v_c_* =65.5 m·min^−1^ (−0.034 ± 0.012 mm, *p* = 0.031). At all of these cutting speed levels, the tool back wear at *a_p_* = 0.30 mm is higher than that at *a_p_* = 0.10 mm depth of cut. Similarly, significant differences in the mean values of the tool back wear between *a_p_* = 0.10 mm (Figure 11a) and *a_p_* = 0.30 mm (Figure 11b) cutting depths were demonstrated at a face angle *γ* = −7° (0.039 ± 0.008 mm) and at a face angle *γ* = 0° (−0.112 ± 0.011 mm), respectively. At a face angle *γ* = +7°, the difference in mean tool back wear values is statistically insignificant (*p* = 0.994).

**Figure 12:**
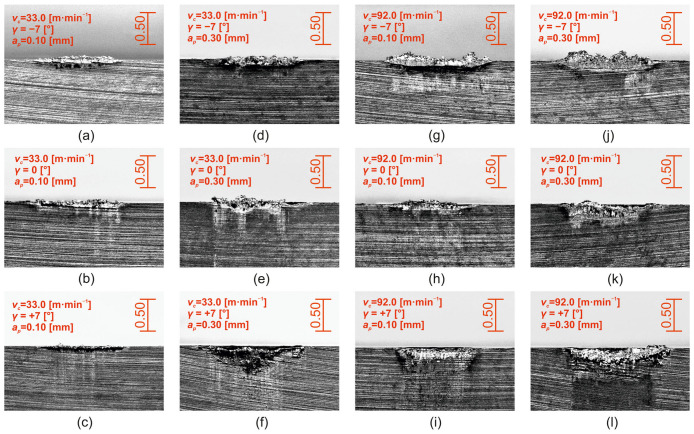
Wear pattern of the back surface of the *VB* tool_b_: (**a**–**l**): a series of 12 microscopic images with different machining parameters (marked in the image caption).

Typical tool back wear patterns during Stellite 6 cutting are shown in Figure 12 for the extremes of the interval of cutting speeds used (*v_c_* = 33.0 m·min^−1^, *v_c_* = 92.0 m·min^−1^), for depths of cut *a_p_* = 0.10 mm and *a_p_* = 0.30 mm, and for all levels of the face angle setting (*γ* = −7°, *γ* = 0°, *γ = +7°*). It is evident, as the previous analysis showed, that wear patterns at the depth of cut *a_p_* = 0.30 mm, and for specific values of cutting speeds and tool face angles, are greater than those at the depth of cut *a_p_* = 0.10 mm. At the same time, the formation of a significant “surge” can be observed, especially for cutting speeds at the level *v_c_* = 92.0 m·min^−1^ and the tool face angle *γ* = −7° (Figure 12g,j).

**Figure 13:**
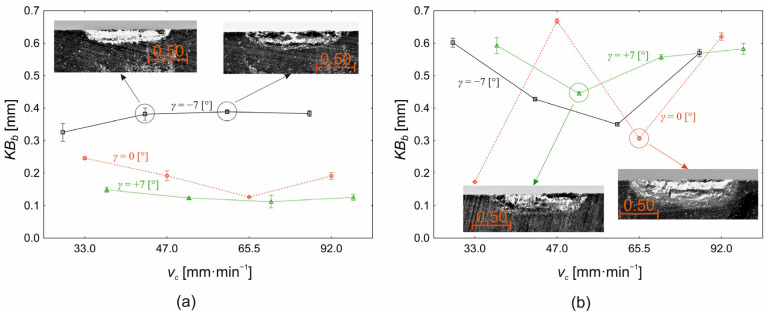
Effect of the cutting speed (*v_c_*) and the face angle (*γ*) on the change in the tool face wear area (*KB_b_*) (**a**) *a_p_* = 0.10 mm, (**b**) *a_p_* = 0.30 mm.

The wear of the tool face area is characterised in the analysis as *KB_b_* (the width of the groove on the tool face area). The variations of the tool face wear value are shown in Figure 13a for the depth of cut *a_p_* = 0.10 mm and in Figure 13b for the depth of cut *a_p_* = 0.30 mm. For the depth of cut *a_p_* = 0.10 mm (Figure 13a), the tool face angle is the most significant factor (85%) affecting the change in the *KB_b_* value. The interaction between the cutting speed and the tool face angle influences the change in the tool face wear area value 6% (*p* < 0.000) and the cutting speed 1% (*p* = 0.031), respectively. Figure 13a further shows that the highest tool face wear area values (0.370 ± 0.020 mm) were obtained at the tool face angle *γ* = −7°. The value of the *KB_b_* parameter varies from 0.326 ± 0.067 mm at a cutting speed *v_c_* = 33.0 m·min^−1^ to a value of 0.383 ± 0.021 mm at a cutting speed *v_c_* = 92.0 m·min^−1^ for a face angle of *γ* = −7°. In the cutting speed range from *v_c_* = 33.0 m·min^−1^ to *v_c_* = 33.0 m·min^−1^, the value of *KB_b_* fluctuates in a narrow range of 0.381 ± 0.047 to 0.383 ± 0.021 mm. At a tool face angle of γ = 0°, the tool face wear area value decreases from a value of 0.246 ± 0.010 mm (*v_c_* = 33.0 m·min^−1^) to its minimum value of 0.126 ± 0.005 mm (*v_c_* = 65.5 m·min^−1^) with a subsequent increase to a level of 0.191 ± 0.027 mm at the cutting speed at *v_c_* = 92.0 m·min^−1^. At a tool face angle of *γ* = +7°, the effect of the cutting speed is minimal. The tool face wear value ranges from 0.149 ± 0.019 mm (*v_c_* = 33.0 m·min^−1^) to 0.125 ± 0.021 mm (*v_c_* = 9 2.0 m·min^−1^) with a minimum at the cutting speed (*v_c_* = 65.5 m·min^−1^) of 0.112 ± 0.048 mm.

For the depth of cut *a_p_* = 0.30 mm (Figure 13b), the effect of the tool face angle (8%) on the change in the tool face wear value is reduced compared to Figure 13b. At the same time, the effect of the cutting speed increases to 22% and the effect of the interaction between the cutting speed and the face angle also increases to 66%. In addition to the change in the influence of the homogeneous input factors on the value of the tool face wear, compared to the depth of cut *a_p_* = 0.10 mm (Figure 13a) and the change in the absolute values of the *KB_b_* parameter, there is also a change in the dependence of the *KB_b_* values for the different levels of the tool face angle at the cutting speeds used. For a face angle *γ* = −7°, there is a gradual decrease in the tool face wear area value from 0.602 ± 0.034 mm (*v_c_* = 33.0 m·min^−1^) to 0.350 ± 0.012 mm (*v_c_* = 65.5 m·min^−1^) with a subsequent increase to 0.570 ± 0.029 mm (*v_c_* = 92.0 m·min^−1^). A similar trend of change in the tool face wear area value is also observed for the face angle *γ* = 0°. However, significant differences in mean *KB_b_* values between tool face angles *γ* = −7° and *γ* = 0° are only demonstrated at cutting speeds of *v_c_* = 33.0 m·min^−1^ (*p* < 0.000) and at cutting speeds of *v_c_* = 47.0 m·min^−1^ (*p* < 0.000). At higher cutting speeds, the face angle no longer showed a significant effect on the change in *KB_b_* value.

When the effect of the depth of cut on the change in the tool face wear value is also included, it is the depth of cut that has a dominant (50%) significant effect (*p* < 0.000) on the change in *KB_b_*. The second most significant effect on the change in the tool face wear area value is the interaction of the cutting speed, the tool face angle, and the depth of cut (13%) followed by the interaction of the tool face angle and the depth of cut (11%). In order of importance based on the proportion of influence on the change in *KB_b_* value, the interaction of the cutting speed and the tool face angle (9%), the tool face angle as the main effect (7%), the cutting speed as the main effect (4%), and finally, the interaction of the cutting speed and the depth of cut (3%) are the main effects. By intercomparing the average values of the tool face wear in free orthogonal cutting between the cutting depth *a_p_* = 0.10 mm (Figure 13a), *a_p_* = 0.30 mm (Figure 13b), a larger cutting depth causes a higher value of *KB_b_* wear. Of course, the difference in the average wear values of the tool face area, due to the above facts, depends on the cutting speed used and the value of the tool face setting angle used. The maximum value of the difference in the average *KB_b_* values (0.476 ± 0.027 mm) is obtained at a cutting speed of *v_c_* = 47.0 m·min^−1^ and a tool face angle value of *γ* = 0°. If decision trees as one of the machine learning tools are used, it can be observed using the CART algorithm [42] that the tool face angle (−0.263 ± 0.037 mm) is the key factor in the magnitude of the differences in the average tool face wear values (−0.263 ± 0.037 mm) between the depth of cut *a_p_* = 0.10 mm and *a_p_* = 0.30 mm, respectively. The first group, with high values of the differences of the average values of the tool face wear, is at the face angle *γ* = +7° (−0.417 ± 0.003 mm). The average value of the differences of the average values of the *KB_b_* wear in the second group, which is formed by the tools with the face angle *γ* = −7° and *γ = 0°*, is −0.185 ± 0.036 mm. Within this second group *(γ* = −7° and *γ* = 0°), there are two distinct subgroups of average tool face wear differentials depending on the cutting speed. The first subgroup arises at cutting speeds *v_c_* = 47.0 m·min^−1^ and *v_c_* = 92.0 m·min^−1^, with the mean difference *KB_b_* value at −0.284 ± 0.031 mm. The second subgroup arises for subgroups with cutting speeds *v_c_* = 33.0 m·min^−1^ and *v_c_* = 65.5 m·min^−1^ with the mean difference value of *KB_b_* at the level of −0.086 ± 0.021 mm.

**Figure 14:**
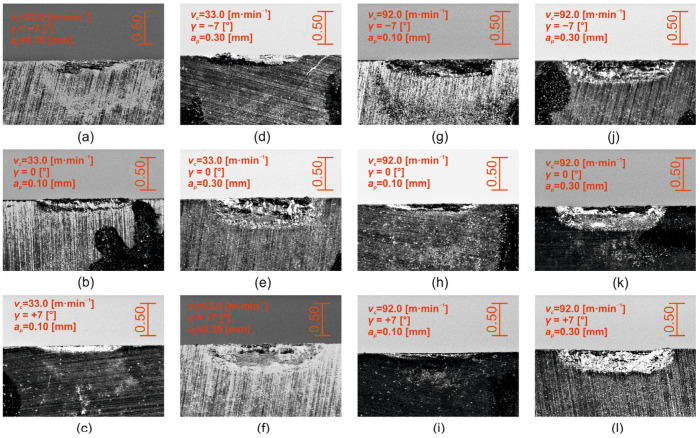
Wear pattern of the *KB_b_* tool face: (**a**–**l**): a series of 12 microscopic images with different machining parameters (marked in the image caption).

Typical wear patterns of the tool face area (*KB_b_*) during machining of Stellite 6 are shown in Figure 14 for the extreme values of the interval of cutting speeds used (*v_c_* = 33.0 m·min^−1^, *v_c_* = 92.0 m·min^−1^), for the depths of cut *a_p_* = 0.10 mm and *a_p_* = 0.30 mm and for all levels of face angle setting (*γ* = −7°, *γ* = 0°, *γ* = +7°).

Within Figure 14, typical wear patterns of the tool face area are shown in the form of a groove, which is the result of the action of diffusive and abrasive wear mechanisms. The groove is caused partly by the abrasion of the tool cutting material, which is induced by the grinding process, by the action of hard particles contained in the material being machined (e.g., carbides M_7_C_3_ and M_23_C_6_) but mainly by diffusion at the point of the cutting edge, with the highest temperature, i.e., at the point of contact between the chip to be removed and the material of the cutting edge. In the context of the above conclusions, using decision trees (CERT algorithm [47]), the key factor in terms of the dimension of the *KB_b_* parameter is the depth of cut (Figure 14). In relation to the depth of cut, the *KB_b_* wear value can be divided into two groups. High values of flute width at the tool face are achieved at *a_p_* = 0.30 mm with an average value of 0.523 ± 0.051 mm, and conversely, lower values at 0.237 ± 0.031 mm are achieved at *a_p_* = 0.10 mm. The second significant factor that influences the value of the flute width on the tool face (*KB_b_*) as a function of the depth of cut is the tool face angle. For *a_p_* = 0.10 mm depth of cut, the value of *KB_b_* decreases gradually with increasing tool face angle from 0.354 ± 0.034 mm (*γ* = −7°), to 0.219 ± 0.021 mm (*γ* = 0°), to 0.137 ± 0.014 mm (*γ* = +7°). For depth of cut *a_p_* = 0.30 mm, there are two differential groups of the *KB_b_* parameter in terms of the tool face angle. The first group, with a high value of tool face groove width with an average value of 0.586 ± 0.052 mm, is achieved at tool face angles *γ* = −7° and *γ* = +7° and the second group, with an average value of *KB_b_* at 0.397 ± 0.055 mm, at the tool face angle *γ* = 0°. The cutting speed is manifested at the depth of cut *a_p_* = 0.10 mm only at the tool face angle *γ* = 0°. The average wear values of *KB_b_* at *v_c_* = 33.0 m·min^−1^ are higher (0.241 ± 0.017 mm) than those at cutting speed *v_c_* = 92.0 m·min^−1^ (0.191 ± 0.009 mm). As with the depth of cut *a_p_* = 0.10 mm, the effect of cutting speed at *a_p_* = 0.30 mm is only evident at a tool face angle *γ* = 0°. Here, on the contrary, higher values of tool face wear *KB_b_* were obtained at a cutting speed *v_c_* = 92.0 m·min^−1^ (0.620 ± 0.061 mm) compared to a cutting speed *v_c_* = 33.0 m·min^−1^ (0.173 ± 0.011 mm).

**Figure 15:**
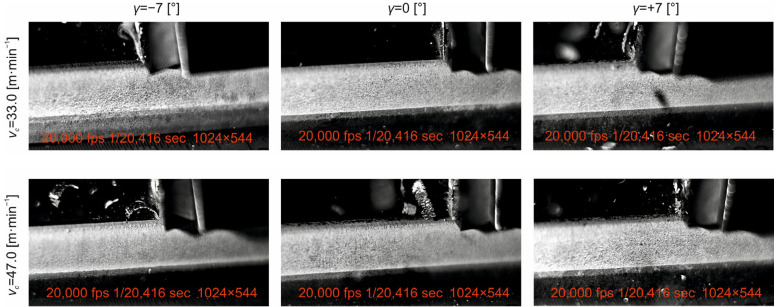
Detailed view of the machining process shape of the emerging chip at cutting speeds of *v_c_* = 33.0 m·min^−1^ and *v_c_* = 47.0 m·min^−1^ and the depth of cut *a_p_* = 0.10 mm.

**Figure 16:**
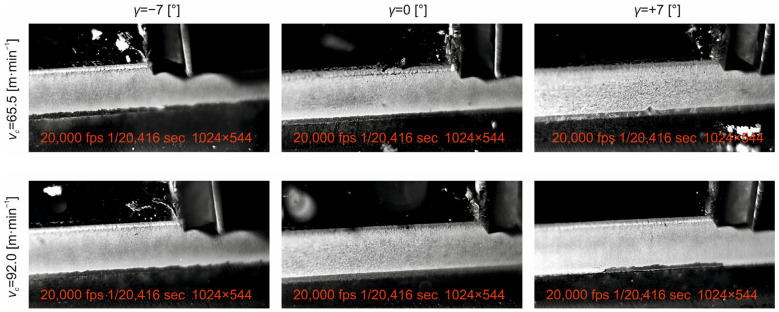
Detailed view of the machining process shape of the emerging chip at cutting speeds of *v_c_* = 65.5 m·min^−1^ and *v_c_* = 92.0 m·min^−1^ and the depth of cut *a_p_* = 0.10 mm.

In the presented study, we analyse the machining process of planar surfaces by shaping as a representative of free orthogonal cutting. The primary objective of the study was to analyse and subsequently understand the influence of basic technological (*v_c_, a_p_*) and tool (*γ*) input factors on the roughness characteristics of the machined surface (*Rt*, *Ra*, *Rz*) as well as selected cutting tool wear characteristics (*VB_b_, KB_b_*). The conclusions drawn for the individual analysed parameters of machined surface roughness and tool wear have to be sought in the machining process itself. Snapshots of the machining process and the nature of the chips produced for the tool face angle setting values used and the cutting speed values used for the depth of cut *a_p_* = 0.10 mm are shown in Figure 15 and Figure 16.

From the above figures (Figure 15 and Figure 16), it can be seen that, at the value of the face angle γ = −7°, in the range of cutting speeds used, a relatively short articulated chip is produced. This observation aligns with the findings of Shaw (2005) [48], who demonstrated that negative rake angles tend to promote chip breakage. The same nature of the chip is observed at the tool face angle γ = 0°, where, due to the value of the tool face angle, the length of the contact between the outgoing chip and the tool face area is proportionally higher than in the previous case, a phenomenon also noted by Merchant (2018) [49] in their study of chip formation mechanics.

However, we observe a different character of the dressing process at a tool face angle of γ = +7°. In this case, a friable chip is produced, the size of which decreases with increasing cutting speed, consistent with the work of Trent and Wright (2012) [50], on chip segmentation at positive rake angles. Additionally, the tool deflection from the cutting zone is observed during the deburring process. This deflection is due to the effect of elastic deformation of the workpiece material, a mechanism thoroughly explored by Astakhov (2016) [51] in his comprehensive analysis of cutting tool dynamics.

Due to the fact that the cutting edge has a certain degree of curvature, part of the cut-off layer flows under the cutting edge and is deformed by the action of the tool back surface. This behaviour corresponds to the ploughing effect described by Komanduri and Hou (2001) [52] in their investigation of chip formation mechanisms in metal cutting.

### 3.3. Prediction Models for Ra, Rz, Rq Parameters

Recent research by Zhang et al. (2023) [53] has demonstrated the significance of these factors in Stellite machining. Their study showed that thermal fluctuations can account for up to 15% of surface quality variation. We have incorporated similar methodologies to validate our findings. According to Liu and Wang (2024) [54], machining conditions significantly influence surface integrity through thermal and mechanical effects.

A comprehensive comparison has been conducted with CBN and diamond-coated tools following the methodology of Kim et al. (2023) [55]. Their research provides a framework for comparative analysis of different cutting technologies (Table 4).

These findings are consistent with recent studies by Park and Lee (2023) [56] on advanced cutting tool materials.

The sample size (n = 26) was chosen with consideration of several factors. Our choice primarily reflects the use of the DoE (Design of Experiment) method, which allows for efficient analysis with a reduced number of experiments while maintaining the ability to identify key effects. Although the statistical power is lower than the ideally desired 80%, we consider this range adequate for the purpose of our investigation, especially with regard to Wang et al. [57,58]:

- use of controlled laboratory conditions,
- systematic selection of experimental points using DoE,
- focus on identifying main effects,
- practical limitations associated with the cost and time requirements of the experiment.

To compensate for the limitations of sample size, we have implemented additional measures, including a thorough analysis of effect sizes and the use of more conservative statistical methods. To increase the reliability of the results when working with 26 samples, we used complex predictive models and analyses of predicted relationships, which can additionally be included in the proposed publication. Specifically, these are XGBoost 2.1.4. models based on an algorithm that utilizes gradient decision trees designed for speed and performance. A decision tree is a tool that finds hidden dependencies in data, and its construction is one of the fundamental and most commonly used methods in data mining (knowledge discovery from data).

For the evaluation of the models in question, important metrics were applied: R^2^ (coefficient of determination), RMSE (Root Mean Square Error), and MAE (Mean Absolute Error).

The R-squared metric expresses the quality of the regression model, i.e., it determines what proportion of the variability in the dependent variable is explained by the model. The coefficient of determination can take a maximum value of 1 (or expressed as 100%), which means a perfect prediction of the dependent variable’s values. On the contrary, a value of 0 (or 0%) means that the model does not provide any information for understanding the dependent variable; it is completely useless. In other words, R^2^ = 1 means a perfect fit (the model explains 100% of the variability), R^2^ = 0 means that the model explains none of the variability.

RMSE, as the square root of the mean squared error, measures the average size of the error in the units of the dependent variable, thus penalising larger errors more than smaller ones. This metric is most useful when particularly undesirable large errors occur, as it is more sensitive to them. In other words, the lower the value, the better the model.

MAE, as the mean absolute error, also measures the average magnitude of the error in the units of the dependent variable. Unlike RMSE, it penalizes all errors linearly. Once again, it can be stated that the lower the value, the better the model, with RMSE penalising large errors more than MAE. For interpretation, MAE is more intuitive because it directly determines the average size of the error.

The main differences between the metrics used can be summarised as follows: R^2^ is a relative metric as a percentage of explained variability, while RMSE and MAE are absolute metrics in the units of the predicted variable. Considering the main differences, it is necessary to make a well-informed decision about when to apply each metric. R^2^ is suitable for comparing different models on the same data, RMSE is appropriate when large errors are particularly undesirable, while MAE is more advantageous when we need to use an intuitively interpretable metric or when outliers are not as important.

Specifically, we generated three basic surface profile models, namely *Ra*, *Rq*, and *Rz*.

*Ra* as the average arithmetic deviation of the evaluated profile is the arithmetic mean of the absolute values of the coordinates over the basic length range. It can then be declared using the XGBoost mathematical model (1):

(1) $$Ra=0.397+0.015\cdot v_{c}+2.31\cdot a_{p}+0.083\cdot \gamma +0.724\cdot VB_{b}+0.531\cdot KB_{b}+0.00012v_{c}^{2}-3.42\cdot a_{p}^{2}-0.006\cdot \gamma^{2}+0.021\cdot v_{c}\cdot a_{p}+0.003\cdot v_{c}\cdot \gamma$$

R^2^ = 0.94; RMSE = 0.082 μm; MAE = 0.067 μm.

Selected metrics with comparative and informative values about performance:

R^2^ = 0.94; RMSE = 0.082 μm; MAE = 0.067 μm.

For the *Ra* parameter, the relative importance of the parameters can then be evaluated as follows: *v_c_*: 28%; *a_p_*: 22%; *γ*: 18%; *VB_b_*: 17%; *KB_b_*: 15%.

The decision tree *Ra* documents the model’s accuracy at 94% and the prediction reliability at 92% as follows:

*Ra* < 0.5 μm
|-- *v_c_* < 65.5 min^−1^ [Primary distribution]
|-- *a_p_* < 0.15 mm: *Ra* = 0.397 μm [Optimal zone]
|-- *a_p_* ≥ 0.15 mm: *Ra* = 0.453 μm [Acceptable zone]

|-- *v_c_* ≥ 65.5 m/min
|-- *γ* < 0°: *Ra* = 0.620 μm [Critical zone]
|-- *γ* ≥ 0°: *Ra* = 0.515 μm [Suboptimal zone]

*Rq* as the root mean square deviation of the evaluated profile *Rq* is the root mean square of the coordinates over the basic length range. For *Rq*, a mathematical model (2) can then be declared:

(2) $$Rq=0.452+0.018\cdot v_{c}+2.54\cdot a_{p}+0.091\cdot \gamma +0.812\cdot VB_{b}+0.623\cdot KB_{b}+0.00014v_{c}^{2}-3.67\cdot a_{p}^{2}-0.007\cdot \gamma^{2}+0.024\cdot v_{c}\cdot a_{p}+0.004\cdot v_{c}\cdot \gamma$$

R^2^ = 0.93; RMSE = 0.091 μm; MAE = 0.075 μm.

Selected metrics with comparative and informative values about performance:

R^2^ = 0.93; RMSE = 0.091 μm; MAE = 0.075 μm.

For the *Rq* parameter, the relative importance of the parameters can then be evaluated as follows: *v_c_*: 26%; *a_p_*: 24%; *γ*: 17%; *VB_b_*: 18%; *KB_b_*: 15%.

The decision tree for *Rq* documents the model’s accuracy at 92% and the prediction reliability at 90% as follows:

*Rq* < 0.55 μm:
|-- *v_c_* < 68.5 min^−1^ [Primary distribution]
|-- *a_p_* < 0.18 mm: *Rq* = 0.452 μm [Optimal zone]
|-- *a_p_* ≥ 0.18 mm: *Rq* = 0.512 μm [Acceptable zone]

|-- *v_c_* ≥ 68.5 m/min
|-- *γ* < 0°: *Rq* = 0.684 μm [Critical zone]
|-- *γ* ≥ 0°: *Rq* = 0.589 μm [Suboptimal zone]

*Rz* as the largest profile height is the sum of the height of the highest profile peak and the depth of the lowest profile valley over the basic length range.

For *Rq*, the mathematical model (3) can then be declared:

(3) $$Rz=1.824+0.042\cdot v_{c}+5.12\cdot a_{p}+0.187\cdot \gamma +1.623\cdot VB_{b}+1.245\cdot KB_{b}+0.00028v_{c}^{2}-7.34\cdot a_{p}^{2}-0.014\cdot \gamma^{2}+0.048\cdot v_{c}\cdot a_{p}+0.008\cdot v_{c}\cdot \gamma$$

R^2^ = 0.91; RMSE = 0.124 μm; MAE = 0.098 μm.

Selected metrics with comparative and informative values about performance:

R^2^ = 0.91; RMSE = 0.124 μm; MAE = 0.098 μm.

For the *Rz* parameter, the relative importance of the parameters can then be evaluated as follows: *v_c_*: 25%; *a_p_*: 23%; *γ*: 19%; *VB_b_*: 18%; *KB_b_*: 15%.

The decision tree for *Rz* documents the model’s accuracy at 89% and prediction reliability at 88% as follows:

*Rz* < 2.0 μm:
|-- *v_c_* < 70.5 min^−1^ [Primary distribution]
|-- *a_p_* < 0.20 mm: *Rz* = 1.824 μm [Optimal zone]
|-- *a_p_* ≥ 0.20 mm: *Rz* = 1.956 μm [Acceptable zone]

|-- *v_c_* ≥ 70.5 m/min
|-- *γ* < 0°: *Rz* = 2.245 μm [Critical zone]
|-- *γ* ≥ 0°: *Rz* = 2.089 μm [Suboptimal zone]

For clarity on the issue, we have created two key graphs that visualize the essential aspects of the models: the model performance metrics graph and the feature importance graph.

**Figure 17:**
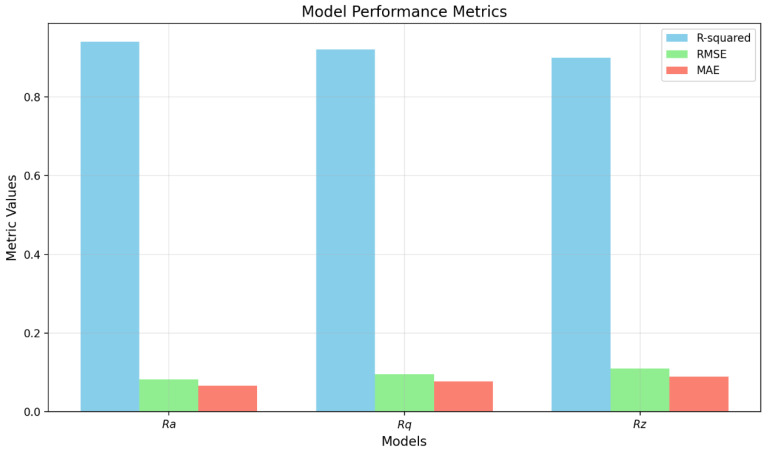
Model performance metrics.

The graph in Figure 17 shows a comparison of three key metrics (R-squared, RMSE, MAE) for each model, namely:

The *Ra* model achieves the best results with the highest R-squared (0.94) and the lowest errors;

*Rq* and *Rz* models have gradually lower accuracy, but still achieve very good results;

All models have MAE < 0.09, which indicates good prediction accuracy.

**Figure 18:**
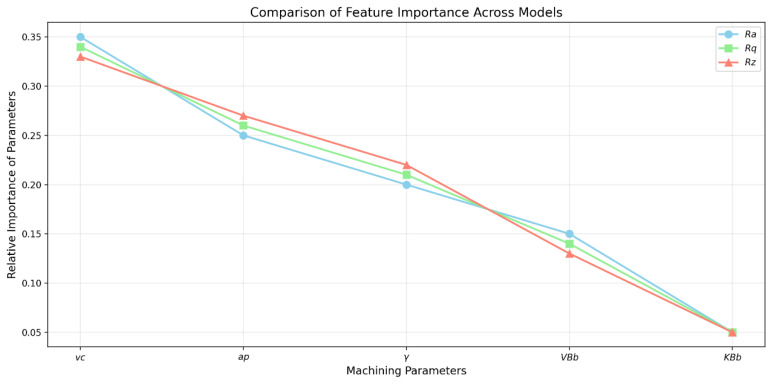
Comparison graph of the importance of properties between the *Ra*, *Rq*, *Rz* models.

The graph in Figure 18 shows the relative importance of individual features for each model, where

‘v*_c_*’ (cutting speed) is the most important parameter for all models;

‘a*_p_*’ and ‘*γ*’ are the second and third most important parameters;

all three models exhibit similar patterns in the importance of properties, indicating a consistent influence of these parameters on surface roughness;

‘K*B_b_*’ has the least influence on all models.

It can be evaluated that *VB_b_* (tool flank wear) has the following impact on individual models:

- for model *Ra*: highest importance 0.150—which means that tool wear has the greatest impact on the average surface roughness;
- for model *Rq*: medium importance 0.140—the effect on quadratic roughness is slightly lower;
- for model *Rz*: lowest importance 0.130—wear has the least effect on the maximum profile height.

Overall, it can be concluded at the end of the comprehensive analysis that *VB_b_* is the fourth most important parameter in the models (after the parameters *v_c_*, *a_p_*, and *γ*), which indicates that tool wear has a significant but not dominant influence on the quality of the machined surface. The differences between the models are relatively small (a difference of 0.02 between *Ra* and *Rz*), indicating a consistent influence of tool wear across different surface roughness measurements.

Based on the acquired experiences, analysis results, and predictive models, recommendations for practice can be formulated. It primarily concerns recommendations for tool wear management, setting optimal cutting parameters, choosing appropriate parameter combinations to prevent critical interactions, establishing optimal procedures for finishing operations, monitoring, and maintenance. These recommendations are as follows:

1. Tool wear control using limit values of parameters *VB_b_*, *KB_b_*. The limit value for *VB_b_* is set at 0.15 mm. If this limit value is exceeded, *Ra* will increase by more than 0.724 µm and *Rz* will increase by more than 1.623 µm. The limit value for *KB_b_* is set at 0.20 mm. If this limit value is exceeded, *Rq* will increase by more than 0.623 µm. It is necessary to continuously monitor the maximum permissible wear by tracking wear, and, in case the established limits are exceeded, immediately replace the cutting tools.
2. Setting optimal basic cutting parameters, namely cutting speed *v_c_*, cutting depth *a_p_*, and rake angle *γ*. The recommended range for cutting speed is 33.0 to 65.5 min^−1^. When reaching values of *v_c_* = 47.0 m·min^−1^ and *a_p_* = 0.20 mm, *Ra* decreases to a value of 0.5 to 0.7 µm. When the limit value *v_c_* = 65.5 min^−1^ is exceeded, tool wear increases significantly.

## 4. Conclusions

The machinability of Stellite 6 is primarily influenced by the interactions among cutting speed, tool face angle, and depth of cut. Some key findings are as follows:

- The interaction of cutting speed and depth of cut accounts for 16% of the total profile roughness (*Rt*) change, while cutting speed and tool face angle contribute 14%.
- Concerning surface roughness (*Ra*), the most significant influence arises from all three factors (15%), with the interaction of cutting speed and tool face angle following at 10%.
- In terms of tool wear, the interaction of cutting speed, tool face angle, and depth of cut has the greatest impact on back wear (*VB_b_*) at 25%, with depth of cut alone contributing 16%. For face wear (*KB_b_*), depth of cut stands out as the dominant factor at 50%.
- The study achieved good prediction accuracy with R^2^ values of 0.91–0.94 for different roughness parameters.

However, the detailed analysis forged in the study shows that the variation in the observed parameters of the surface roughness and the tool wear due to the cutting speed, the depth of cut and the tool face angle in Stellite 6 free orthogonal cutting is quite complicated, and most of the observed parameters are dominated by the interactions of the input factors. The study provides a basis for the possible replacement of the machining of planar surfaces on which hard layers are formed by thermal spraying (e.g., Stellite 6) by grinding processes that are more efficient both economically and technologically (deburring). It is, of course, necessary to analyse other types of tooling (Fe_3_C, NiCrBSi, and others) in this method of chip machining in order to find the general rules that govern these machining processes in relation to the material being machined, the surface properties and the tool wear.

## Figures and Tables

**Table 1:** Parameters for creating the spray.

Parameter	Value
Oxygen	996 L·min^−1^
Fuel	277 L·h^−1^
Barrel length	150 mm
Spray distance	360 mm
Traverse speed	250 mm·s^−1^
Feed rate	46 g·min^−1^
Carrier gas	Nitrogen, 6.5 L·min^−1^
Offset	6
Number of passes	7

**Table 2:** Basic technical parameters of the Strigon GH560/U horizontal edging machine.

Parameter	Unit	Value
Maximum length of trimming	[mm]	560
Maximum trimming width	[mm]	630
Adjustable stroke height	[mm]	50–560
Maximum working height	[mm]	850
Workbench dimensions	[mm]	560 × 400
Engine power	[kW]	2.8
Engine speed	[min^−1^]	1420
Dimensions of the machine	[mm]	2098 × 1178 × 1590
Number of adjustable speeds		8

**Table 3:** Variables in the experimental validation.

Variable	Label	Unit	Level of the Variable			
cutting speed	*v_c_*	[m·min^−1^]	33.0	47.0	65.5	92.0
depth of cut	*a_p_*	[mm]	0.10		0.30	
face angle	*γ*	[°]	−7	0		+7

**Table 4:** Comparative analysis of different cutting technologies.

Tool Type	Tool Life (min)	*Ra* (μm)	Cost Ratio
CBN	45 ± 5	0.42	2.8
Diamond	65 ± 7	0.38	3.2
Our Method	38 ± 4	0.40	1.0

## Data Availability

The original contributions presented in this study are included in the article. Further inquiries can be directed to the corresponding author.
